# Low Numbers of FOXP3 Positive Regulatory T Cells Are Present in all Developmental Stages of Human Atherosclerotic Lesions

**DOI:** 10.1371/journal.pone.0000779

**Published:** 2007-08-22

**Authors:** Onno J. de Boer, Jelger J. van der Meer, Peter Teeling, Chris M. van der Loos, Allard C. van der Wal

**Affiliations:** Department of Pathology, Academic Medical Center, University of Amsterdam, Amsterdam, The Netherlands; Monash University, Australia

## Abstract

**Background:**

T cell mediated inflammation contributes to atherogenesis and the onset of acute cardiovascular disease. Effector T cell functions are under a tight control of a specialized T cell subset, regulatory T cells (Treg). At present, nothing is known about the in situ presence of Treg in human atherosclerotic tissue. In the present study we investigated the frequency of naturally occurring Treg cells in all developmental stages of human atherosclerotic lesions including complicated thrombosed plaques.

**Methodology:**

Normal arteries, early lesions (American Heart Association classification types I, II, and III), fibrosclerotic plaques (types Vb and Vc) and ‘high risk’ plaques (types IV, Va and VI) were obtained at surgery and autopsy. Serial sections were immunostained for markers specific for regulatory T cells (FOXP3 and GITR) and the frequency of these cells was expressed as a percentage of the total numbers of CD3^+^ T cells. Results were compared with Treg counts in biopsies of normal and inflammatory skin lesions (psoriasis, spongiotic dermatitis and lichen planus).

**Principle findings:**

In normal vessel fragments T cells were virtually absent. Treg were present in the intima during all stages of plaque development (0.5–5%). Also in the adventitia of atherosclerotic vessels Treg were encountered, in similar low amounts. High risk lesions contained significantly increased numbers of Treg compared to early lesions (mean: 3.9 and 1.2%, respectively). The frequency of FOXP3^+^ cells in high risk lesions was also higher compared to stable lesions (1.7%), but this difference was not significant. The mean numbers of intimal FOXP3 positive cells in atherosclerotic lesions (2.4%) was much lower than those in normal (24.3%) or inflammatory skin lesions (28%).

**Conclusion:**

Low frequencies of Treg in all developmental stages of human plaque formation could explain the smoldering chronic inflammatory process that takes place throughout the longstanding course of atherosclerosis.

## Introduction

A chronic, T cell mediated inflammatory process determines the growth and development of atherosclerotic lesions [1]–[3]. Although the majority of the inflammatory cells in atherosclerotic lesions are macrophages, up to 20 percent of the cells are T lymphocytes [2], [4]. Moreover, activation of T cells is considered to play an important role in the process of atherosclerotic plaque destabilization which may initiate plaque disruption and the onset of acute coronary syndromes[5]–[7]

In general, the effector functions of activated T cells are under tight control of a special subset of T cells, the regulatory T cells (Treg). Treg play a central role in inducing and maintaining immunologic tolerance and the termination of immune responses. Deficiency or dysfunction of these cells lead to autoimmunity or aggravated pathogen-induced inflammation[8], [9]. An important population of Treg is formed by the naturally occurring Treg, phenotypically characterized by a high constitutive expression of CD25, the alpha chain of the interleukin-2 receptor, and also referred to as CD4^+^CD25^+^ regulatory T cells[10]. In tissues these cells are best identified by their expression of transcription factor FOXP3 (Forkhead Box Protein P3), a member of the forkhead winged helix protein family of transcription factors[11].

Since atherosclerotic plaque inflammation also relates to autoimmunity, likely induced by oxidized LDL epitopes [12], a role for Treg in atherosclerotic inflammatory process could be anticipated. Indeed, experimental studies have shown that adoptive transfer of Treg prevent the development of plaques in mouse models of atherosclerosis[13]–[15]. Furthermore, it was recently shown that the numbers of Treg are decreased in the peripheral blood of patients with acute coronary syndromes[16]. However, the in situ presence of Treg in human atherosclerotic plaques has not yet been investigated.

In the present study we systematically evaluated the distribution of regulatory T cells in all developmental stages of human plaques (type 1 to VI according to the classification of the American Heart Association, AHA) with the use of monoclonal antibodies against regulatory T cell markers FOXP3 and GITR. Cellular specificity of FOXP3 and GITR for T cells was investigated with immunodoublestains visualized with spectral imaging [17]. Moreover, using the AHA classification of lesions, we compared specifically the relative frequency of Treg in early, stable, and high risk (vulnerable and thrombosed) plaques. In order to get insight in the numbers of Treg that participated in atherosclerosis, we compared these data with the Treg counts in normal human skin and in the inflammatory infiltrates encountered in various types of T cell mediated inflammatory skin diseases.

## Materials and Methods

### Specimens

Atherosclerotic vessel fragments were collected from 42 patients undergoing vascular surgical interventions and from 3 patients at autopsy (mean age: 62.1 years±9.5). A total of 69 different diseased vessel fragments were studied from the aorta (n = 26), a.carotis (n = 32, all endarterectomies), and a.femoralis (n = 11). All specimens were formalin fixed and paraffin embedded. After preparation of hematoxylin-eosin and Elastic van Gieson stained sections, plaques were categorized according to the American Heart Association classification of atherosclerotic lesions[18]: type1: initial lesion (n = 7); type II, fatty streak (n = 10); type III, proatheroma (n = 7); type IV, atheroma (n = 7); type Va, fibroatheroma (n = 9); type Vb, calcified plaque (n = 8), type Vc, fibrotic plaque (n = 9) and type VI, complicated plaques (plaques with thrombus formation or intraplaque hemorrhage, n = 12). For additional comparisons in the present study, these lesions were pooled into 3 groups: early lesions (AHA type I, II, and III); fibrosclerotic lesions (stable plaques, AHA type Vb and Vc) and ‘high risk’ plaques (vulnerable and thrombosed plaques, AHA type IV, Va and VI).

As reference materials we used segments of mammary arteries, which are known to be relatively resistant to the development of atherosclerotic plaques. The segments were obtained from 10 patients (mean age: 65.2 years±9.7) undergoing coronary artery bypass surgery, in which mammary artery was used in the bypass graft, and the remainder of the artery was send to the pathology lab. Of these, 36 cross sectional segments were collected, which showed a normal arterial wall (n = 30) or early atherosclerotic lesions (AHA type 1 (n = 5) and AHA type 2 (n = 1)).

Frequencies calculated from atherosclerotic tissue were compared with a similar study performed on normal and inflamed skin specimens, performed using identical methods and techniques [17]. In that study distribution of Treg was investigated on T cells in normal human skin (n = 14), psoriatic skin (n = 13), spongiotic dermatitis (n = 13) and lichen planus (n = 12).

Tissue samples used in this study were from the “AMC-vesselbank”, a collection of well documented vessel fragments obtained after vascular surgical interventions or autopsy[19], [20], and oral informed consent was obtained from all participants or relatives included in this study. Acquisition of tissue samples was approved by the institutional medical ethical committee and the study was conducted according to the Declaration of Helsinki Principles.

### Immunohistochemistry

Immunohistochemical single and double stains were performed on 5 µm thick sections prepared from formalin fixed, paraffin embedded tissue, as previously described [17]. The following antibodies were used: rabbit monoclonal anti CD3 (SP7, Labvision, Freemont, CA), mouse monoclonal anti FOXP3 (clone 236A/E7; Abcam, Cambridge, UK), goat polyclonal anti-GITR (R&D Systems Europe Ltd), mouse monoclonal anti CD20 (Labvision), mouse monoclonal anti CD68 (PGM1, Dako) and mouse monoclonal anti CD138 (Dako). As a second step the following reagents were used: Rabbit anti-goat (Dako), Poly-AP anti-mouse (Immunovision Technologies, Brisbane, USA) and Envision+anti-rabbit (Dako). As chromogens we used DAB+ and Liquid Permanent Red Kit (both from Dako). Spectral imaging, a computer assisted optical technique allowing a detailed analysis of double stained cells was performed as previously described[17].

#### Quantification

Quantification of FOXP3 and GITR positive T cells in atherosclerotic tissue was performed in single-stained serial sections. The centre section was stained for CD3, whereas the two adjacent sections were stained for FOXP3 and GITR, respectively. Frequency of FOXP3 and GITR positive cells were determined by taking a digital overview image of each CD3-stained section at a low power magnification (10×2). In each of these images 4 areas in the region of interest (intima or adventitia) with the highest density of CD3 positive cells were manually marked, using image analysis software (Image Pro Plus, vs. 5.01, Media Cybernetics, Silver Spring, MD). Next, these selected areas were retraced at high power (10×20) magnification and in each set of adjacent GITR, CD3, and FOXP3-stained sections digital images were captured of these spots. The total numbers of positive cells in interrelated images were counted using the “manual tag” option of Image Pro Plus, and the numbers of GITR and FOXP3 positive cells were expressed as percentage of CD3 positive cells.

#### Statistical analysis

Statistical differences between the different inflammatory conditions were evaluated by One way Anova, followed by post-hoc Tukey test. P<0.05 was considered statistically significant. Statistical analysis was performed using the SPSS software package (Chicago, IL, USA, vs. 11.5 for Windows).

## Results

### 1. Distribution of CD3, FOXP3, and GITR in normal human vessel fragments and atherosclerotic tissue

**Figure 1:**
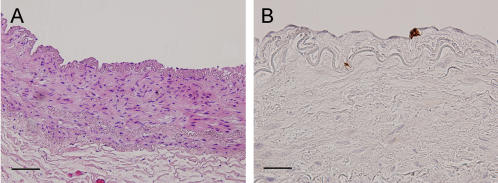
Histology of normal human artery. Hematoxylin&eosin overview (A, bar = 200 µm) and detailed CD3 (B, bar = 50 µm) staining of a normal human (mammary) artery. CD3 positive T cells are rare in normal human intima.

First, we investigated the distribution of T cells and Tregs in normal human vessel fragments. Thirty different control vessel fragments from the mammary artery, a vessel know to be relatively resistant for the development of atherosclerotic plaques were studied. T cells were virtually absent in the normal arterial intima: in the 30 specimens of mammary arteries we investigated, a total of 22, scattered T cells were encountered. Similar low counts of T cells were found in the adventitial tissue surrounding normal vessel fragments. On average, approximately 1.4 T cell/mm^2^ was encountered. In none of the specimens FOXP3 or GITR positive cells were encountered. Figure 1 shows a representative example of a normal mammary artery, stained with H&E (A) and CD3 (B).

**Figure 2:**
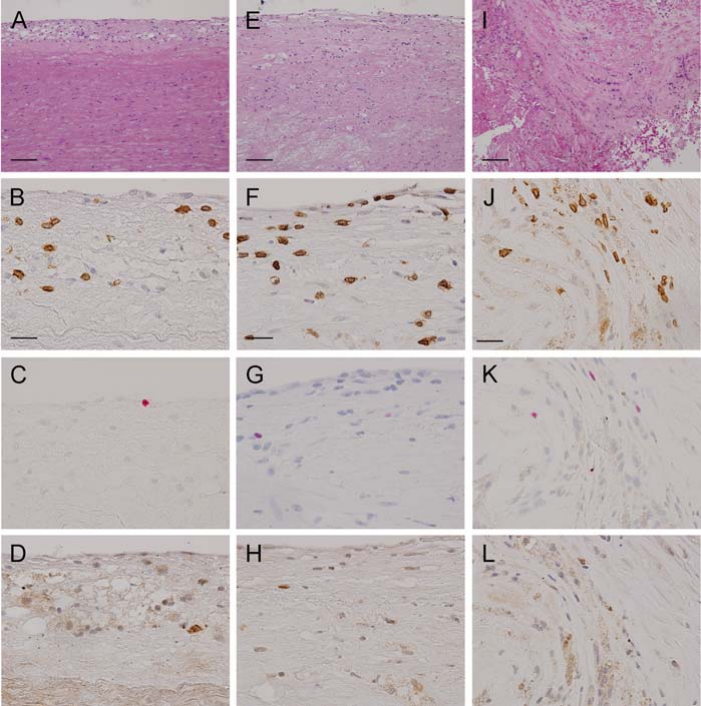
Histology of different types of atherosclerotic arteries. Hematoxylin&Eosin overview (A, E, I, bar = 200 µm) and detailed CD3 (B, F, J, bar = 50 µm), FOXP3(C, G, K) and GITR(D, H, L) stains of three different types of atherosclerotic lesions: AHA class II (A–D), AHA class Va (E–H) and AHA class VI (I–L). CD3 positive T cells are frequently present in atherosclerotic tissue, but Treg are scarce.

Representative histological examples of the intima of atherosclerotic vessels, i.e an early lesion (AHA class II), an advanced lesion (AHA class Va) and a recently thrombosed plaque (AHA class VI) are illustrated in Figure 2, where sections stained with hematoxylin&eosin, CD3, FOXP3, and GITR are shown. CD3 positive T cells were found in the intima in lesions from all stages of plaque development. The numbers of CD3 positive T cells increased with the plaque severity. FOXP3 and GITR positive cells were observed in the intima in all stages of plaque development but their numbers were, compared to CD3 very low.

In 29 of the specimens (13 early-, 4 fibrosclerotic-and 11 high risk plaques) adventitial tissue was present which could be studied as well. In all these specimens mononuclear infiltrates were present which were composed of T cells, B cells, macrophages and plasma cells. FOXP3 and GITR positive cells were also encountered in these specimens but, similar to atherosclerotic intima, their numbers were low. However, focal accumulations of GITR expressing cells were observed in 4 high risk plaques. This was typically the case in those specimens with follicular lymphoid infiltrates, which are known to be associated with advanced atherosclerosis.

**Figure 3:**
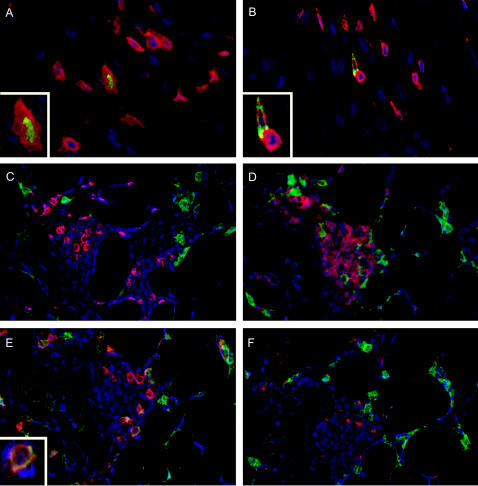
Double staining images after spectral imaging. A: FOXP3 (green) and CD3 (red) in atherosclerotic intima. Nuclei are depicted in blue. Inset shows double stained cell. Note nuclear staining of transcription factor FOXP3 and cytoplasmatic CD3 positivity; B: GITR (green) and CD3 (red) in atherosclerotic intima. Inset shows one double and one single stained cell; C: GITR (green) and CD3 (red) in atherosclerotic adventitia. GITR positive cells do not co-express CD3. D: GITR (green) and CD20 (red). No double stained cells. E: GITR (green) and CD138 (red). Inset shows double stained cells. F: GITR (green) and CD68 (red). No double stained cells.

Immunodoublestains were performed to verify the phenotype of the FOXP3^+^ and GITR^+^ cells in atherosclerotic tissue. In atherosclerotic intima, FOXP3^+^-and GITR^+^ cells were always CD3 positive (see figure 3A and B for representative examples), indicating that all these FOXP3 and GITR positive cells are T cells. In atherosclerotic adventitia FOXP3 was always co-expressed with CD3. However, GITR positive cells in the lymphoid adventitial infiltrates in some high risk plaques were not only T cells, but additionally CD138^+^ plasma cells were GITR positive (fig 3D). CD20^+^ B cells and CD68^+^ macrophages were GITR negative (Figure 3D and 3F, respectively).

### 2. Frequency of FOXP3 and GITR in atherosclerotic intima

**Figure 4:**
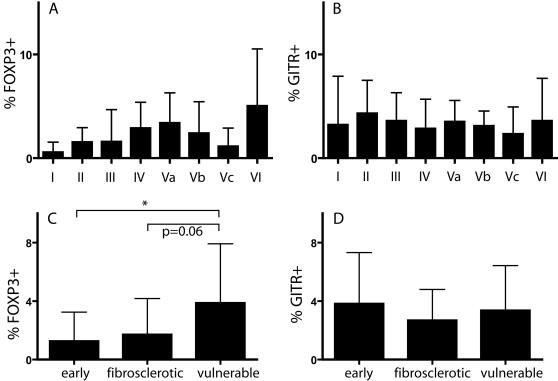
Frequency of FOXP3 and GITR positive T cells in atherosclerotic tissue. Values are expressed as a percentage (±SD) of the total number of T lymphocytes. Plaques were categorized according to the AHA classification (A and B, respectively) or categorized as early, fibrosclerotic and vulnerable lesions (C and D, respectively). *: P<0.05

After having assessed that all FOXP3 and GITR positive T cells in atherosclerotic intima are indeed T cells, we counted the numbers of CD3, FOXP3 and GITR positive T cells and expressed the numbers of FOXP3 and GITR positive cells in these lesions as a percentage of the total number of T cells (figure 4A). The frequency of FOXP3 positive T cells varied from 0.5% in AHA class I-to 5% in class VI lesions. Using the AHA classification, we found no significant differences between the different groups. However, when comparing ‘high risk’ lesions with early lesions, we observed that the frequency of FOXP3 positive cells T cells was significantly increased from 1.2 to 3.9.% (Figure 4C). The frequency of FOXP3 positive T cells in advanced lipid rich lesions was also higher compared fibrosclerotic plaques (3.9 vs 1.7%), but this difference was just not significant (p = 0.06).

The mean frequency of GITR positive cells is illustrated in Figure 4B. The mean frequency of intimal GITR positive T cells was in the range of 2.3 (AHA Vc) to 4.3% (AHA II). No significant difference in the frequency of GITR positive T cells between the different types of lesions was found (Figures 4B and 4D).

### 3. Frequency of FOXP3^+^ and GITR^+^ cells compared with atherosclerotic adventitia, normal and inflammatory skin

**Figure 5:**
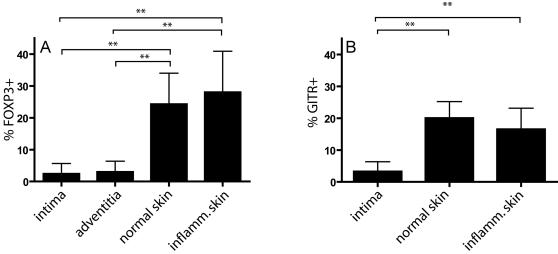
Treg in atherosclerotic tissue compared to normal skin and inflammatory dermatoses. Frequency of FOXP3 (A) and GITR (B) positive T cells in atherosclerotic and dermal specimens. Values are expressed as percentage (±SD) of the total number of T lymphocytes. The frequency of Treg in atherosclerotic intima and adventitia are significantly lower compared to both normal skin as well as a panel of inflammatory dermatoses (psoriasis, spongiotic dermatitis and lichen planus). **: P<0.005

Knowing the frequency of FOXP3 and GITR positive cells in atherosclerotic intima, we next wanted to know the frequency of these cells in the adventitia, and under inflammatory conditions in a different tissue. Figure 5A shows that the mean frequency of FOXP3 positive T cells in atherosclerotic intima and adventitia was similar (all types of lesions were pooled). However, the frequency of FOXP3 positive T cells in normal, as well as in inflammatory skin lesions was significantly higher, 24 and 28%, respectively. Similar results were observed with GITR (figure 5B): the frequency of GITR positive cells was significantly higher in normal and inflammatory skin lesions compared to atherosclerotic intima.

## Discussion

Regulatory T cells play a central role in inducing and maintaining immunologic tolerance and the termination of immune responses, and deficiency or dysfunction of these cells lead to autoimmunity or aggravated pathogen-induced inflammation[8], [9]. Also in atherosclerotic disease the presence and function of regulatory T cells may have important implications, because of their potential suppressive effects on the in situ inflammatory response. In the present study we investigated the presence of naturally occurring regulatory T cells in normal and atherosclerotic vessels by studying the presence of FOXP3 and GITR positive T cells. In normal intima T cells are virtually absent. T cells and Treg are present in atherosclerotic plaques, but the frequency of the latter is low (0.5–5%). Despite these relatively low numbers, related to the total number of T cells present in the plaque, we found that high risk plaques (AHA IV, Va and VI) contain significantly more FOXP3 positive T cells compared to early lesions (AHA 1, II and III). It is known that the onset of acute coronary syndromes is associated with changes in the inflammatory response in these lesions, including a shift in phenotype of intraplaque T cells: the frequency of activated T lymphocytes is significantly increased in unstable lesions [5], [21]. Furthermore, it is has been shown that the onset of acute coronary syndromes is associated with the antigen driven, oligoclonal proliferation of certain T cell subsets[6]. It appears that as a result of such an increase in T cell mediated inflammatory activity, also the frequency of Treg increases in these unstable lesions.

At present, most studies on Treg in relation to (auto) immunity have been performed on peripheral blood Treg. Peripheral blood Treg are relatively easily detectable by their high constitutive expression of CD25 (CD4^+^CD25^high^ subset). In humans, decreased frequency of peripheral blood CD4^+^CD25^high^ Treg have been linked to different types of autoimmune diseases like rheumatoid arthritis, type-1 diabetes, multiple sclerosis and systemic lupus erythematosus [22]–[25]. Recently it was shown that the frequency of Treg is also decreased in the peripheral blood of patients with acute coronary syndromes[16], [26]. Our study is the first describing (low numbers) of Treg also inside human plaques.

In the present study we used FOXP3 and GITR to analyze the presence of Treg in situ. GITR is directly involved in maintaining immunological tolerance by GITR-GITRligand interactions [27], [28]. However, recently activated T lymphocytes may also express GITR, and therefore the expression of GITR by T lymphocytes should be taken with caution. Still, in the present study we found that, apart from the adventitia in advanced atherosclerotic plaques, the distribution of GITR and FOXP3 were comparable, and the results obtained with GITR were in line with the observations obtained with FOXP3. Transcription factor FOXP3 is now generally considered as the most reliable antigen to identify naturally occurring Treg *in situ* [17], [29], [30]. Only recently reliable monoclonal antibodies specific for FOXP3 became available[31], and therefore there are not many data available yet on the distribution or frequency of Treg in solid tissues. In the present study we observed that the mean frequency of FOXP3 positive Treg in atherosclerotic intima, but also in atherosclerotic adventitia was in the range of the 0.5–5% of the total number of CD3 positive T cells. By contrast, in a previous study [17], performed under identical methodological conditions, we found that the frequency of FOXP3 positive T cells in normal skin and a panel of inflammatory skin diseases was in the range of 25–30%, which is significantly higher compared to the situation in atherosclerotic intima and adventitia. It thus appears that, at least compared to normal and various T cell mediated inflammatory skin diseases, the frequency of Treg in atherosclerotic tissue is very low. This raises two important questions: first, what the reason for this low frequency of Treg in atherosclerotic tissue, and the second, does this have biological and/or clinical implications.

Recently, it was shown that oxidized lipoproteins inhibited FOXP3 expression and Treg function of mouse effector cells *in vitro* [15]. Thus, it could be that oxidized lipids, present already in the intima in the earliest stages of atherosclerosis, locally inhibit FOXP3 expression and Treg function *in vivo*. Indeed, we found that the frequency of Treg is not only relatively low in advanced, but also in early lesions. Similarly, the low frequency of FOXP3^+^ Treg in the adventitia could be the result of plaque derived lipoproteins, transported via Vasa vasorum microvessels to the adventitia. On the other hand, it is also possible that other mechanisms are responsible for the low numbers of Treg, like a decreased homing of Treg into vascular tissue. Still, it also important to note the frequency of Treg may be highly variable between different solid tissues, and so far we only compared (inflammatory) skin and atherosclerotic vessels.

The second important question that should be addressed concerns the biological and clinical implications of this low frequency of Treg in atherosclerotic tissue. In this respect it is first important to emphasize again that both experimental, as well as studies in humans have clearly shown that T cells and T cell activation are very important in development of atherosclerotic lesions, and the onset of acute cardiovascular syndromes [32]. Furthermore, it has been shown that increasing the numbers of Treg in atherosclerosis prone (ApoE-/-) mice by means of adoptive transfer leads to smaller atherosclerotic lesions[13]–[15], implicating that Treg do appear capable modifying plaque volume in vivo. These observations, taken together with the findings on the in situ expression of Treg in human plaques of the present study make it tempting to speculate that such low umber of Treg do have implications could be explain the smoldering chronic inflammatory process in the longstanding course of atherosclerosis.
